# Mutant Cx30-A88V mice exhibit hydrocephaly and sex-dependent behavioral abnormalities, implicating a functional role for Cx30 in the brain

**DOI:** 10.1242/dmm.046235

**Published:** 2021-01-26

**Authors:** Nicole M. Novielli-Kuntz, Eric R. Press, Kevin Barr, Marco A. M. Prado, Dale W. Laird

**Affiliations:** 1 Department of Anatomy and Cell Biology, The University of Western Ontario, London, ON, Canada N6A 5C1; 2 Department of Physiology and Pharmacology, The University of Western Ontario, London, ON, Canada N6A 5C1; 3 Robarts Research Institute, The University of Western Ontario, London, ON, Canada N6A 5K8

**Keywords:** Connexin, Connexin 30, Gap junctions, Mice, Mutant

## Abstract

Connexin 30 (Cx30; also known as *Gjb6* when referring to the mouse gene) is expressed in ependymal cells of the brain ventricles, in leptomeningeal cells and in astrocytes rich in connexin 43 (Cx43), leading us to question whether patients harboring *GJB6* mutations exhibit any brain anomalies. Here, we used mice harboring the human disease-associated A88V Cx30 mutation to address this gap in knowledge. Brain Cx30 levels were lower in male and female Cx30^A88V/A88V^ mice compared with Cx30^A88V/+^ and Cx30^+/+^ mice, whereas Cx43 levels were lower only in female Cx30 mutant mice. Characterization of brain morphology revealed a disrupted ependymal cell layer, significant hydrocephalus and enlarged ventricles in 3- to 6-month-old adult male and female Cx30^A88V/A88V^ mice compared with Cx30^A88V/+^ or Cx30^+/+^ sex-matched littermate mice. To determine the functional significance of these molecular and morphological changes, we investigated a number of behavioral activities in these mice. Interestingly, only female Cx30^A88V/A88V^ mice exhibited abnormal behavior compared with all other groups. Cx30^A88V/A88V^ female mice demonstrated increased locomotor and exploratory activity in both the open field and the elevated plus maze. They also exhibited dramatically reduced ability to learn the location of the escape platform during Morris water maze training, although they were able to swim as well as other genotypes. Our findings suggest that the homozygous A88V mutation in Cx30 causes major morphological changes in the brain of aging mice, possibly attributable to an abnormal ependymal cell layer. Remarkably, these changes had a more pronounced consequence for cognitive function in female mice, which is likely to be linked to the dysregulation of both Cx30 and Cx43 levels in the brain.

## Abstract (precis)

**Summary:** This study reveals that adult mice harboring a disease-linked Cx30 mutant exhibit morphological changes in the brain that result in behavioral abnormalities that are more pronounced in female mice.

## INTRODUCTION

Understanding how connexin dysregulation and gene mutations lead to disease is complex, given that there are 21 family members in humans and that most cells express two or more of these channel-forming proteins (Sohl and Willecke, 2004). The canonical role of connexins in most cells is to assemble into hexameric channels that traffic to the cell surface and dock to connexin hexamers from an adjoining cell to create gap junction (GJ) channels (Laird, 2006). The resulting GJ channels allow for the selective passage of numerous small molecules, ranging from ions to secondary messengers to metabolites, in a process called gap junctional intercellular communication (GJIC) (Goldberg et al., 2002). In some cases, particularly in pathologies, connexin hexamers can function as hemichannels at the cell surface, allowing for highly regulated small molecule exchange between the cytoplasm and the extracellular environment (Goodenough and Paul, 2003; Leybaert et al., 2017; Srinivas et al., 2018). Often, both GJ and hemichannel function become dysregulated when connexin gene mutations produce mutant connexins that exhibit gain-of-function or loss-of-function properties, providing a molecular basis for disease (Kelly et al., 2015; Kuang et al., 2020; Laird, 2008; Laird and Lampe, 2018; Srinivas et al., 2018). At present, there are nearly 30 diseases, which present in multiple organs, linked to gene mutations in no less than half of the connexin gene family (Laird et al., 2017). Although skin disorders constitute nearly a dozen of these diseases (Srinivas et al., 2018), it remains extremely difficult to predict where and when a connexin gene mutation will present as a pathological anomaly in a tissue manifesting in disease.

Connexin 30 (Cx30; also known as *GJB6* when referring to the human gene) is best understood as one of several connexins found in the epidermis of the skin and exhibits plentiful expression in the organ of Corti in the inner ear (Di et al., 2001; Forge et al., 2003). Accordingly, mutations in the human gene (*GJB6*) lead to skin disease and hearing loss (Common et al., 2002; Smith et al., 2002). In the skin, *GJB6* mutations result in the rare autosomal dominant disorder hidrotic ectodermal dysplasia 2 (HED2), also known as Clouston syndrome (Avshalumova et al., 2013; Fraser and Der Kaloustian, 2001; Smith et al., 2002). HED2 is characterized by thinning wiry hair, patches of alopecia, hyperkeratosis of the palms, which take on a cobblestone appearance, and nail dystrophy (Fraser and Der Kaloustian, 2001; Lamartine et al., 2000). In humans, no less than four amino acid changes in Cx30 are linked to HED2 (Baris et al., 2008; Jan et al., 2004; Smith et al., 2002; Zhang et al., 2003). In the organ of Corti, Cx30 is assembled into large gap junctions in the diverse cell network that supports hair cell survival, and several *GJB6* gene mutations have been documented to cause hearing loss (Berger et al., 2014; Common et al., 2002). *GJB6* is also expressed in the brain, raising the possibility that patients harboring heterozygous *GJB6* gene mutations might suffer from cognitive deficiencies that remain clinically unreported or become evident only during aging.

The most studied of the *GJB6* disease-causing mutations has been a mutant in which alanine at position 88 is substituted with a valine (p.A88V) (Bosen et al., 2014; Kelly et al., 2019; Lukashkina et al., 2017; Zhan et al., 2020). The autosomal dominant p.A88V mutation in Cx30 is considered to be the cause of HED2, because it has been identified in no less than seven families encompassing at least 15 individuals (Essenfelder et al., 2004; Smith et al., 2002; Zhang et al., 2003). The p.A88V mutant appears to assemble into leaky hemichannels, which disrupts Ca^2+^ and ATP homeostasis found in the skin (Essenfelder et al., 2004; Kuang et al., 2020).

In order to understand the consequences of the A88V mutation *in vivo*, Cx30-A88V knock-in mice were generated (Bosen et al., 2014). These mice exhibited oversized sebaceous glands and relatively mild palmoplantar hyperkeratosis (Bosen et al., 2014). In a recent elegant study, Kuang et al. (2020) developed a novel anti-Cx30 antibody that could reverse the skin pathology mediated by leaky hemichannels in homozygous Cx30-A88V mutant mice (Kuang et al., 2020), raising the profile of potential connexin-targeted therapeutics. Homozygous mice harboring the A88V mutation exhibited a second pathology of low-frequency hearing loss (Bosen et al., 2014). Intriguingly, these same mutant mice were protected from high-frequency, age-related hearing loss, suggesting that dysregulated Cx30 in the cochlea can provide a physiological advantage (Bosen et al., 2014). Further characterization of the hearing loss protection found in Cx30-A88V mice revealed that age-dependent outer hair cell loss was greatly reduced in mutant mice (Bosen et al., 2014; Kelly et al., 2019; Lukashkina et al., 2017). Notably, Cx30 is amply expressed in astrocytes, ependymal cells, leptomeningeal cells and brain pericytes (Abudara et al., 2014; Dere et al., 2003; Mazaré et al., 2018; Nagy et al., 2001) (see also http://mousebrain.org/genesearch.html). This raised the question as to whether Cx30-A88V mutant mice might develop additional morbidities during aging related to brain development and function.

Here, we investigated Cx30 in the brains of 3- to 6-month-old male and female Cx30-A88V mutant mice and compared them with littermate controls. We found that homozygous Cx30-A88V mice exhibited lower levels of Cx30 and sex-dependent effects on co-expressed connexin 43 (Cx43). Homozygous mutant mice presented with increased brain weight, increased ventricular size and hydrocephalus. Strikingly, despite similar structural brain changes, homozygous female mutant mice exhibited worse behavioral outcomes, including deficits in learning. These experiments illuminate how mutant Cx30 can impact the mammalian brain and contribute to pathology.

## RESULTS

### Female mutant mice exhibit greater reductions in connexin levels compared with males

**Fig. 1.**
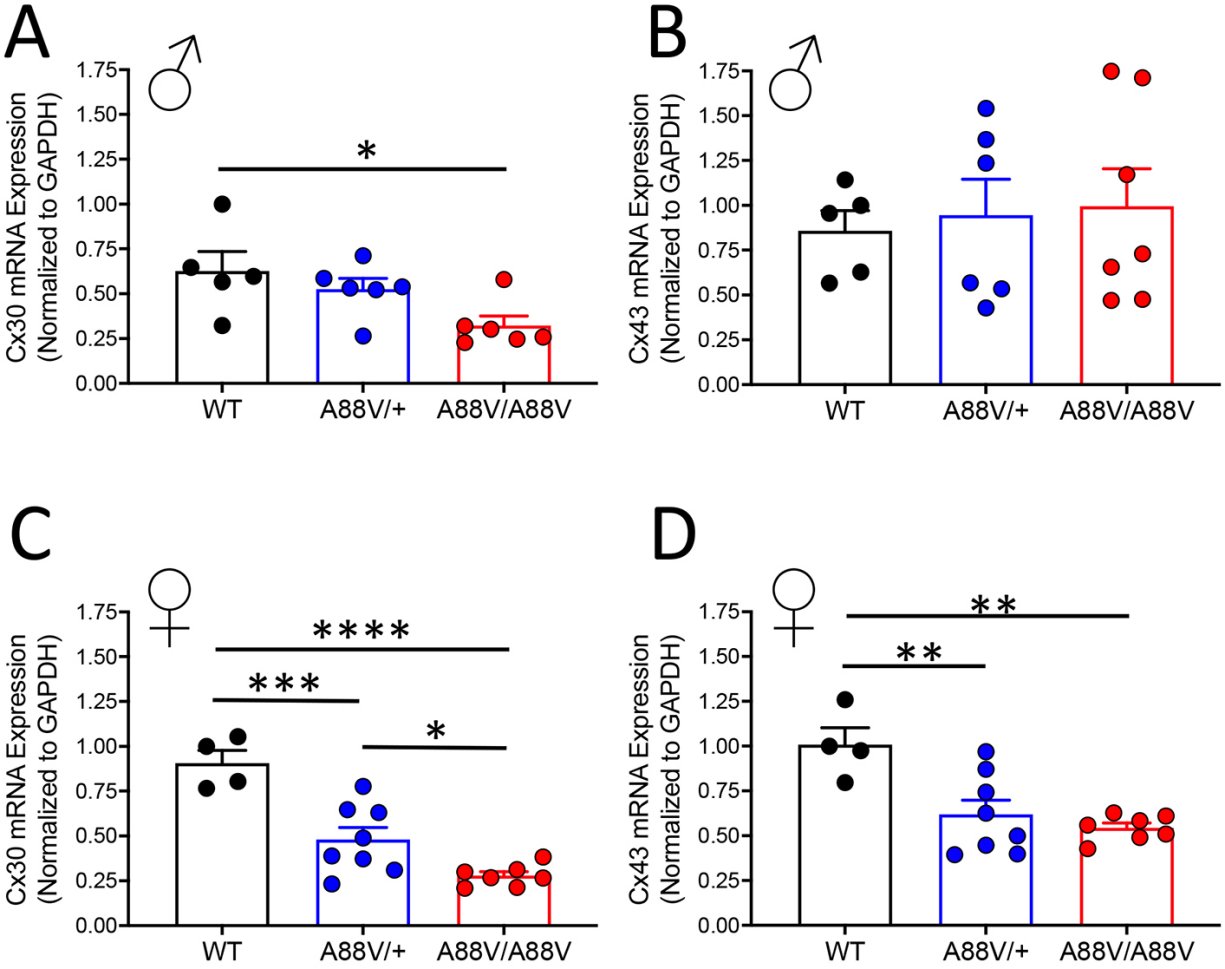
**Cx30 and Cx43 mRNA levels in whole-brain lysates from male and female WT, heterozygous and homozygous mutant mice.** (A-D) mRNA expression of Cx30 (A,C) and Cx43 (B,D) in whole-brain lysate of male and female mice, respectively (*P<0.05, **P<0.01, ***P<0.001, ****P<0.0001; one-way ANOVA). Connexin mRNA expression levels are normalized to GAPDH.

**Fig. 2.**
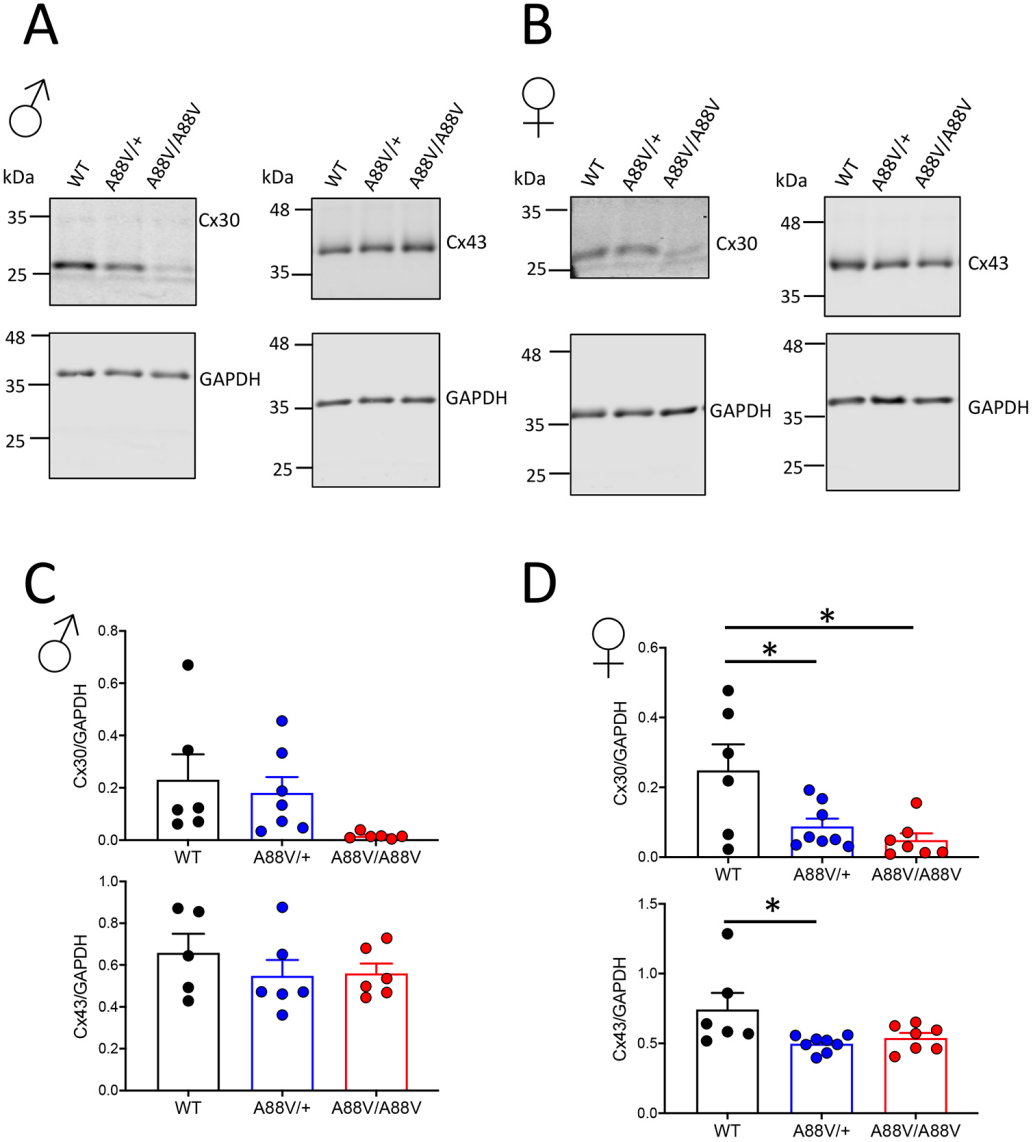
**Cx30 and Cx43 protein levels in whole brain lysates from male and female WT, heterozygous and homozygous mutant mice.** (A-D) Representative western blots and protein expression analysis of Cx30 and Cx43 in whole brain lysates of male (A,C) and female (B,D) mice (*P<0.05; one-way ANOVA). Immunoblotting for GAPDH was used as a loading control. Data are expressed relative to GAPDH expression.

Both Cx30 and Cx43 are expressed in the brain, most notably as the connexins forming gap junctions between astrocytes, although Cx43 is far more plentiful compared with Cx30 (Nagy et al., 2001). To investigate whether the presence of the A88V mutation in Cx30 affected either Cx30 and/or Cx43 levels in the brains of 3- to 6-month-old mutant mice, their expression and localization were assessed. In males, Cx30 mRNA levels were decreased in Cx30^A88V/A88V^ compared with wild-type (WT) mice (Fig. 1A), whereas Cx43 mRNA levels were similar between groups (Fig. 1B). In female mice, however, Cx30 mRNA levels were lower in Cx30^A88V/+^ mice and further reduced in Cx30^A88V/A88V^ mice compared with WT mice (Fig. 1C). Furthermore, both heterozygous and homozygous mutant female mice displayed reduced Cx43 mRNA levels compared with WT mice (Fig. 1D). At the protein level, Cx30 was low in Cx30^A88V/A88V^ mice (Fig. 2A,B), although Cx43 protein expression was unchanged between the male mouse genotypes (Fig. 2A,C). In female mice, Cx30 was also less abundant in Cx30 mutant mice compared with WT mice (Fig. 2B,D), and Cx43 was statistically less abundant only in Cx30^A88V/+^ mice compared with WT mice (Fig. 2D).

### Homozygous mutant mice develop hydrocephaly

**Fig. 3.**
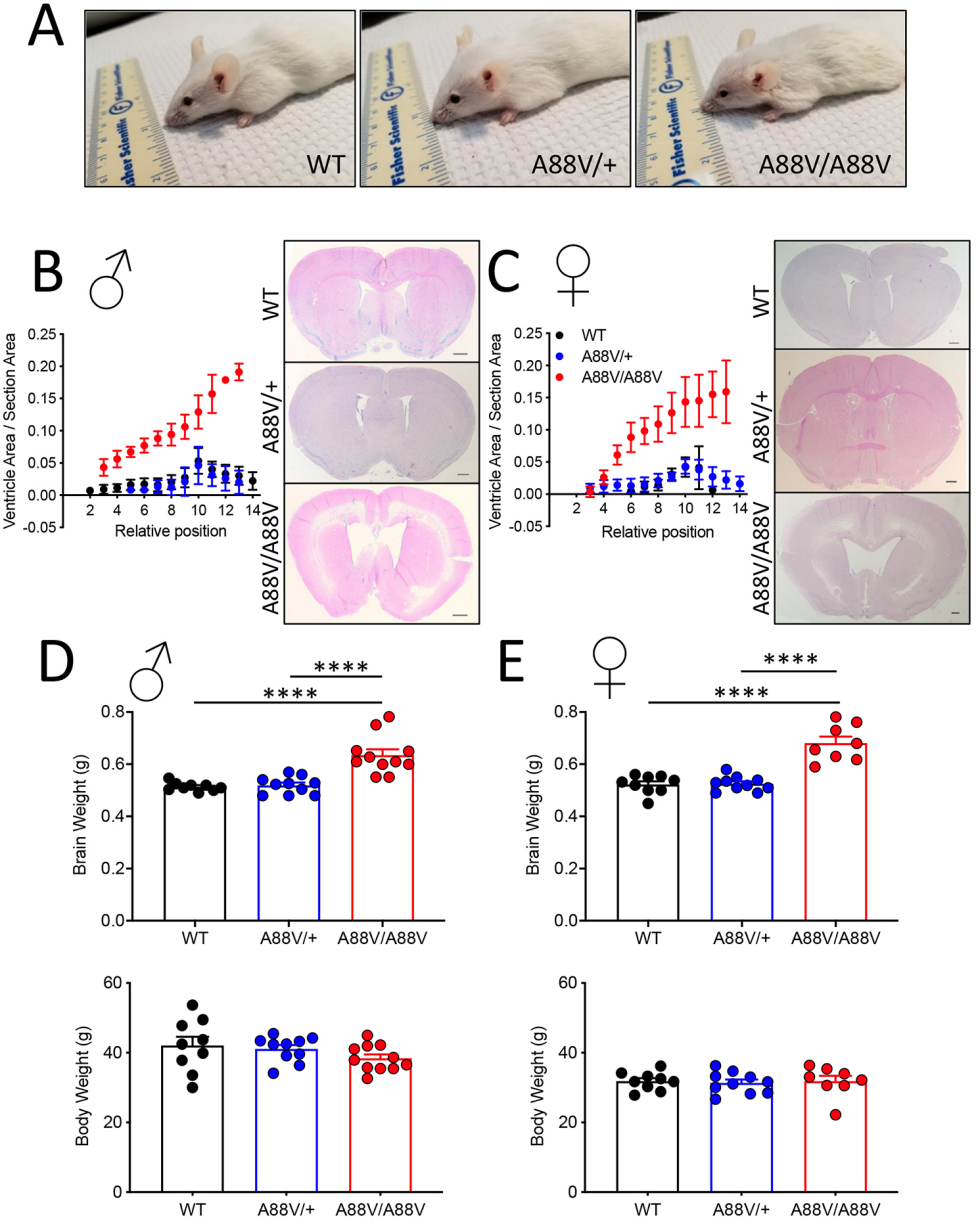
**Male and female homozygous mutant mice exhibit greater brain weight and enlarged ventricular volume indicative of hydrocephaly.** (A) Morphological representation of WT, heterozygous and homozygous mutant male mice. (B,C) Histological representation of coronal brain sections at comparable posterior positions in the three genotypes of both male (B) and female (C) mice. Note the enlarged ventricular area of homozygous mutant mice compared with WT and heterozygous mice. Sections were stained with Hematoxylin and Eosin. Scale bars: 0.5 mm, *n*=3. (D,E) Male (D) and female (E) homozygous mutant mice display significantly greater brain weight than WT and heterozygous mutant mice despite similar body weights (****P<0.0001; one-way ANOVA, Tukey's post test).

To determine whether the alterations in Cx30 and Cx43 affected the brain of aging mice, we examined brain weight and gross morphology in Cx30^+/+^, Cx30^A88V/+^ and Cx30^A88V/A88V^ male and female adult mice (Fig. 3A). Analysis of coronal brain sections demonstrated enlarged ventricular areas (indicative of volume changes) in both male and female Cx30^A88V/A88V^ mice compared with sex-matched WT and heterozygote mutant mice (Fig. 3B,C). Furthermore, both male and female Cx30^A88V/A88V^ mouse brain weights were significantly greater than those found in Cx30^+/+^ or Cx30^A88V/+^ mice, despite similar body weight (Fig. 3D,E). These findings clearly indicate that Cx30^A88V/A88V^ mice had developed hydrocephaly.

**Fig. 4.**
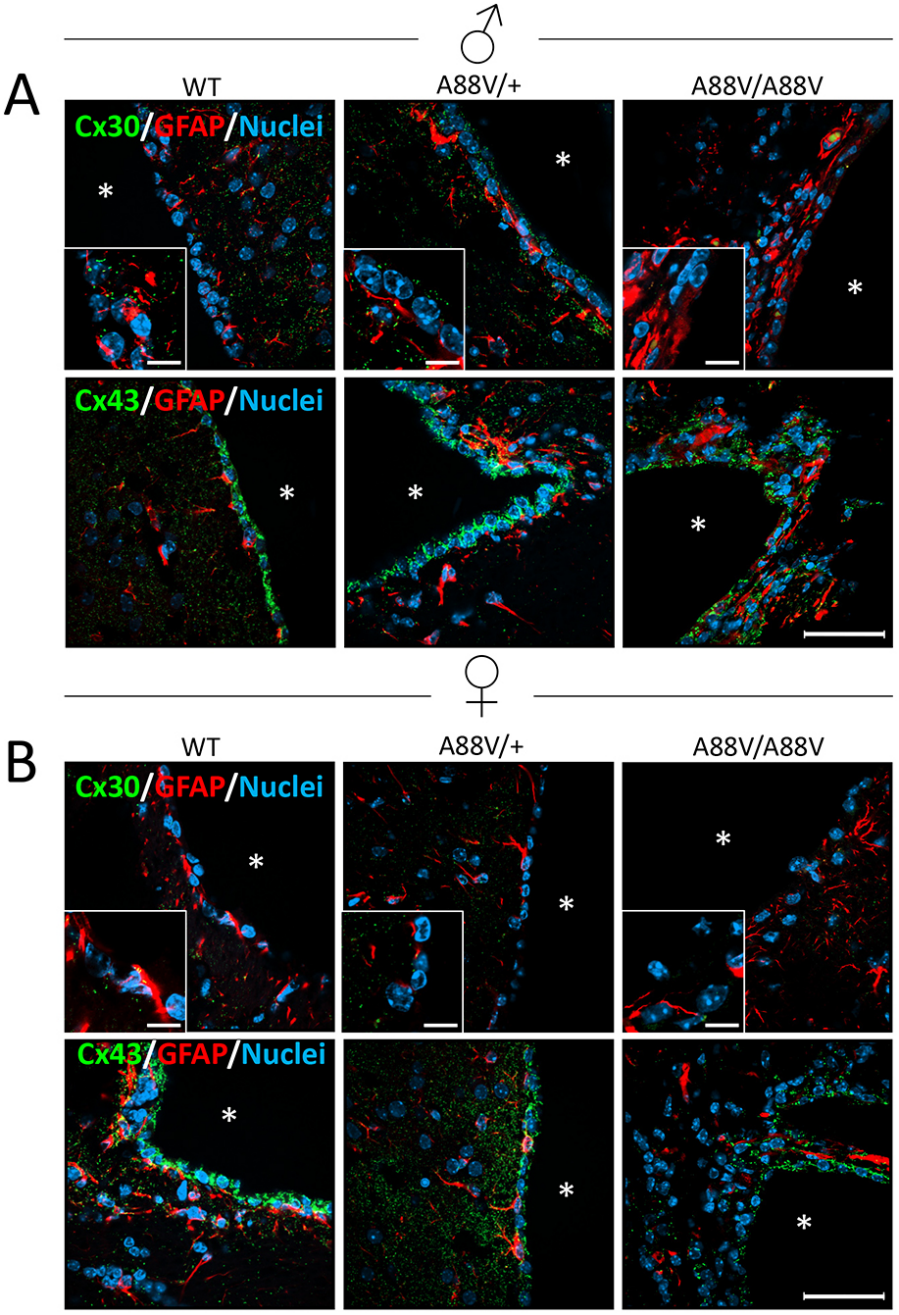
**Cx30 and Cx43 expression in ventricle-lining ependymal cells.** (A,B) Representative immunofluorescence images of male (A) and female (B) mice reveal the localization and relative expression profile of Cx30 (green), Cx43 (green) and GFAP (red; astrocyte marker) in coronal brain sections at the level of the ventricles. Cx43 is expressed in the ependymal cell layer in abundance across all genotypes, whereas Cx30 is seen to a lesser extent, especially in homozygous mutant mice (A88V/A88V). Insets represent a magnified area of the image to highlight the position of the connexins. Asterisks indicate the ventricle space. Scale bars: 50 µm (20 μm for insets).

Given that Cx30 has been shown to be expressed in ependymal cells lining the brain ventricles (Kunzelmann et al., 1999), and these cells contribute to cerebral spinal fluid production, we assessed whether Cx30 and Cx43 levels and localization were changed in ependymal cells of mutant mice (Fig. 4). In both male (Fig. 4A) and female (Fig. 4B) mouse brains, Cx30 was detectible as green puncta along the ependymal cells lining the ventricles (denoted by asterisks), but to a much lesser extent than the plentiful levels of Cx43. Owing to the low level of Cx30 detected and the loss of a clearly intact ependymal cell lining of the ventricles (Fig. 4), it was difficult to determine convincingly whether homozygous mutant mice had less Cx30 compared with WT mice, although our western blot analysis might suggest that this was the case in female mutant mice. However, it is important to recognize that the level of Cx30 might also be reduced in astrocytes and leptomeningeal cells of mutant mice, but this is not possible to discern clearly in these immunofluorescence images.

**Fig. 5.**
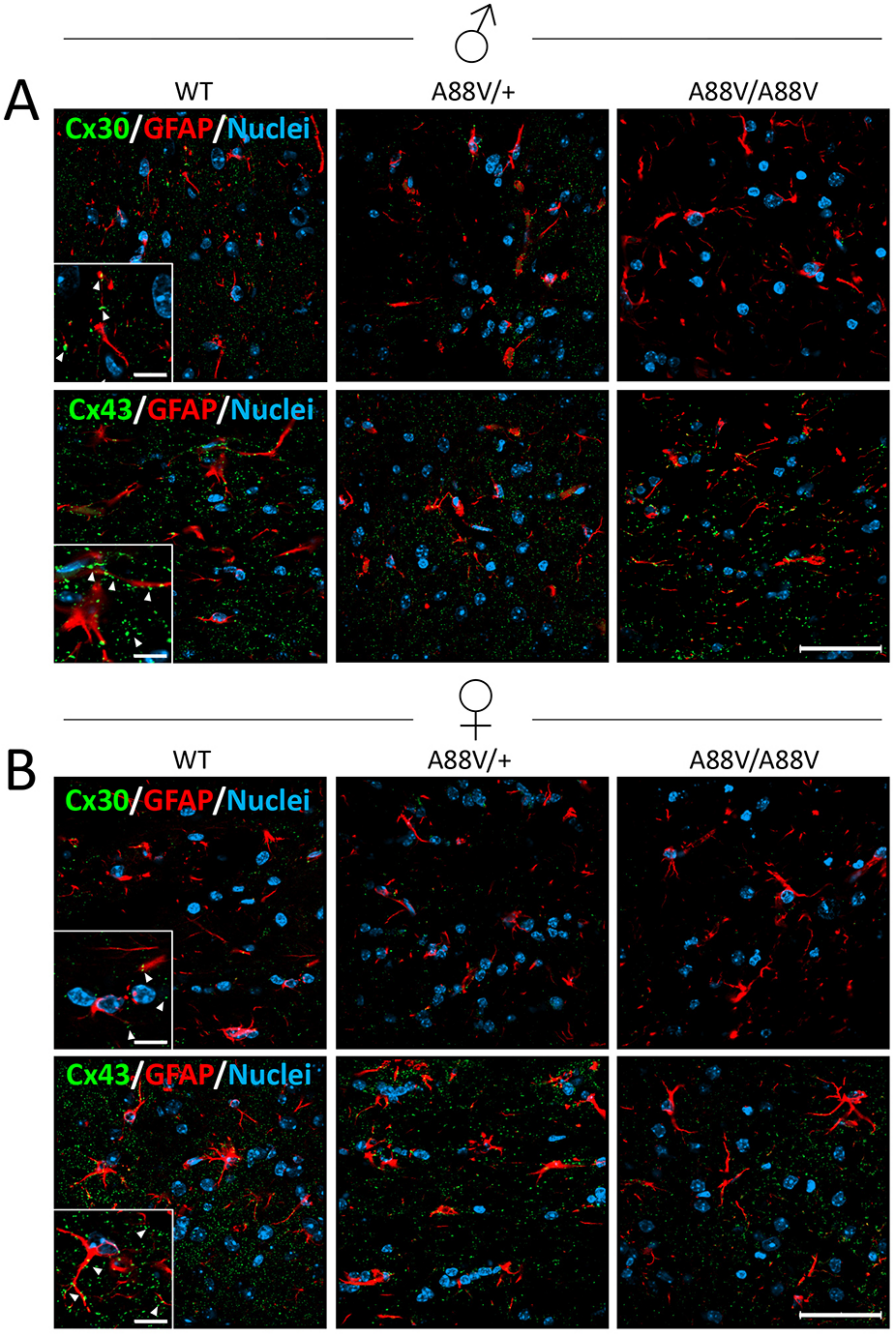
**Cx30 and Cx43 are localized in proximity to astrocytes.** (A,B) Representative immunofluorescence images of male (A) and female (B) mice reveal low Cx30 (green) levels in coronal brain sections (especially in A88V/A88V mutant mice), whereas Cx43 (green) is abundantly expressed in proximity to astrocytes, as denoted by GFAP (red). Insets represent a magnified area of the image to highlight the position of the connexins (arrowheads). Scale bars: 50 µm (20 μm for insets).

To assess the localization of Cx43 and Cx30 in the whole brains of male and female WT and mutant mice, coronal sections were double immunolabeled for connexin and glial fibrillary acidic protein (GFAP) to demarcate the location of astrocytes (Fig. 5). Although Cx43 was abundant, little Cx30 immunoreactivity was detected in astrocytes from male (Fig. 5A) or female (Fig. 5B) mice. Hence, the Cx30 localization and expression patterns in brain tissue were generally concurrent with the reduced Cx30 mRNA and protein levels seen in homozygous mutant mice.

### Behavioral studies as a measure of cognitive function

**Fig. 6.**
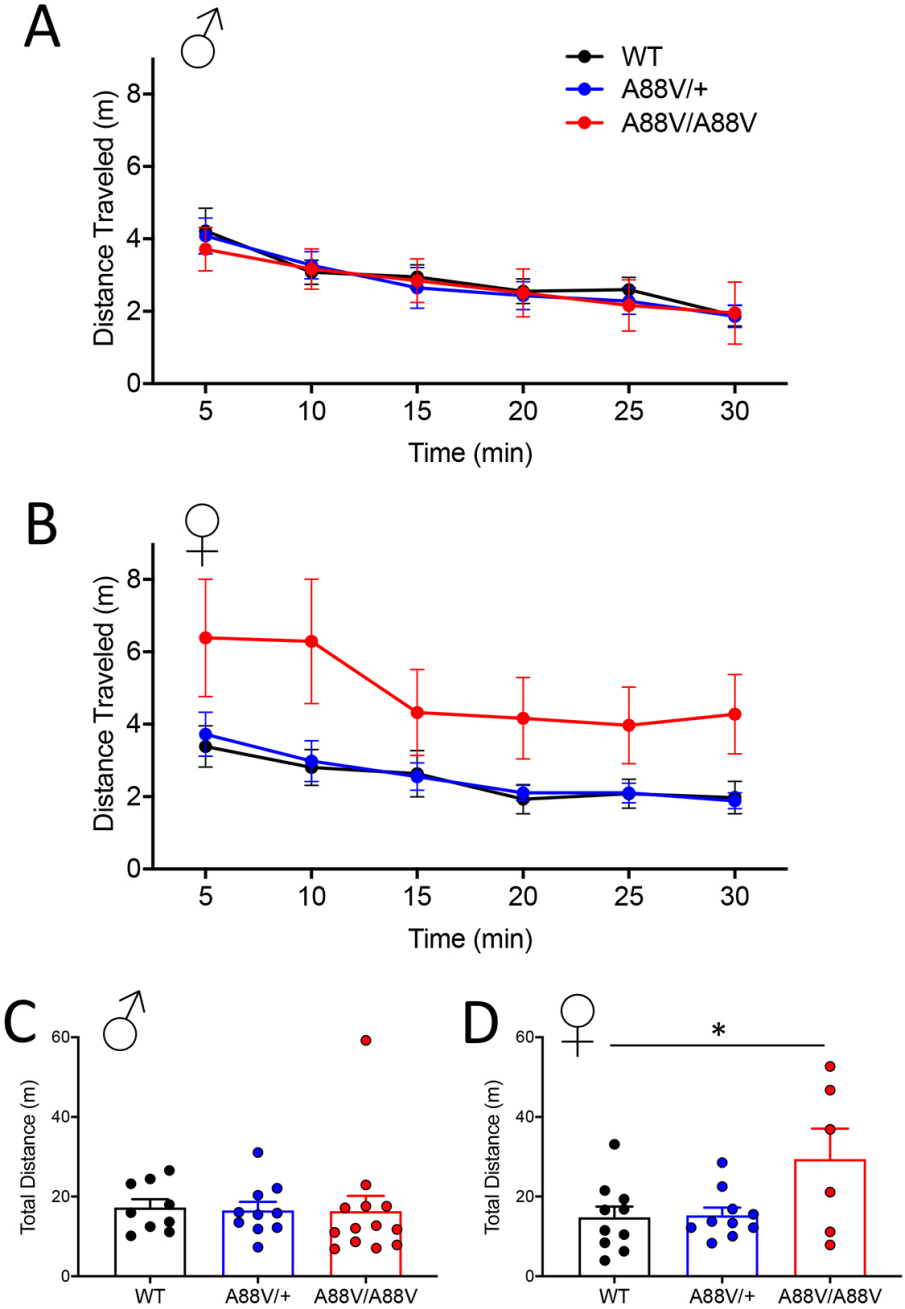
**Female homozygous mutant mice exhibit greater locomotor activity during open field testing.** (A,B) Male (A) and female (B) WT, heterozygous and homozygous mutant mice were subjected to an open field test to assess locomotor activity for a period of 30 min. Note that female homozygous mutant mice displayed more variable locomotor activity in the new environment. (C,D) Male mice from all genotypes traveled similar total distances (C), whereas female homozygous mutant mice traveled further than WT mice (*P<0.05; one-way ANOVA, Tukey's post test) (D).

Given that Cx30^A88V/A88V^ mice exhibited sex-dependent changes in Cx43 and enlarged ventricles surrounded by an apparently disrupted ependymal cell layer, it was important to determine whether heterozygous mutant mice, which model human disease, or homozygous mutant mice present with functional behavioral deficits. To assess general locomotor behavior, mice were examined using the open field test. All male mouse genotypes traveled similar distances over 30 min in an open environment (Fig. 6A,C). Interestingly, however, homozygous female mutant mice displayed more variability in locomotor activity and greater total distance traveled, suggesting increased exploratory behavior in a new environment (Fig. 6B,D).

**Fig. 7.**
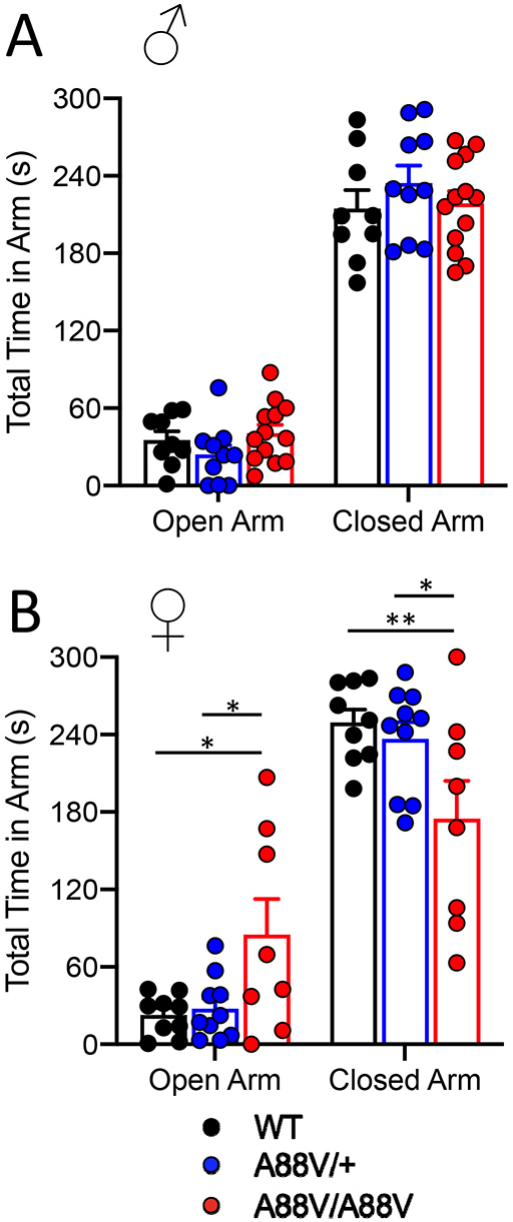
**Female homozygous mutant mice exhibit greater exploratory behavior in the elevated plus maze.** (A) Male WT, heterozygous and homozygous mutant mice spent similar amounts of time in the open and closed arms of the elevated plus maze. (B) Compared with WT and heterozygous mice, female homozygous mutant mice spent more time in the open arm and less time in the closed arm of the elevated plus maze (*P<0.05, **P<0.01: two-way ANOVA with Tukey's post test).

To test the behavior of Cx30 mutant mice further, they were studied with the elevated plus maze (Fig. 7). All male mice spent the most time in the closed arm and far less time in each of the open arms (Fig. 7A). Notably, despite greater variability than WT and Cx30^A88V/+^ mice, female Cx30^A88V/A88V^ mice spent significantly more time in the open arms and less time in the closed arms (Fig. 7B), supporting the notion that Cx30^A88V/A88V^ female mice exhibit increased exploratory behavior, even in a threatening environment (open arms).

**Fig. 8.**
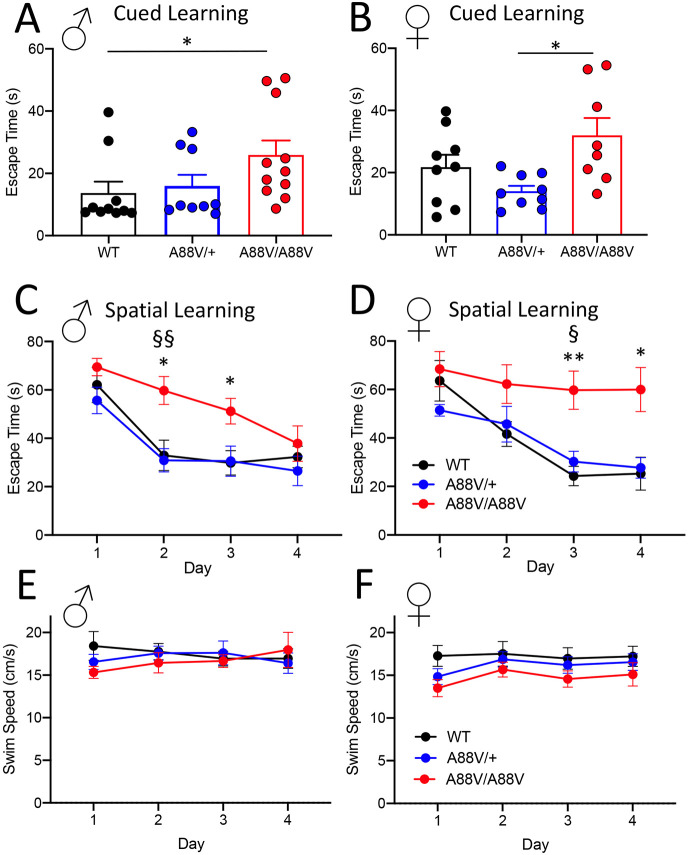
**Homozygous mutant mice perform poorly in the Morris water maze testing model.** (A,B) Both male (A) and female (B) homozygous mutant mice took longer to reach the flagged platform when subjected to the Morris water maze test (*P<0.05; one-way ANOVA). (C) Male homozygous mutant mice also demonstrated delayed learning of the hidden platform location at days 2 and 3, but exhibited similar escape times to other genotypes by day 4. (D) Female homozygous mutant mice demonstrated greatly impaired learning of the hidden platform location, as demonstrated by the lack of improvement in escape time over a 4-day period, unlike WT and heterozygote mutant mice. (E,F) Male (E) and female (F) mice displayed similar swimming speeds during the spatial learning phase of the Morris water maze (*P<0.05, **P<0.01, for WT versus A88V/A88V; ^§^*P*<0.05, ^§§^*P*<0.01, for A88V/+ versus A88V/A88V; two-way repeated measures ANOVA with Tukey's post test).

Lastly, male and female mice were subjected to the Morris water maze to assess spatial learning. For the cued test, where the position of the escape platform was clearly marked with a mounted flag, both male and female homozygous mutant mice took longer to reach the platform (Fig. 8A,B). However, in the case of the female homozygous mutant mice, statistical significance was achieved only when compared with heterozygous mutant mice, probably owing to a wide range of variance in the WT control group (Fig. 8B). During the spatial acquisition phase, on average, all male genotypes gradually reduced the time to escape over the 4 days of testing. However, Cx30^A88V/A88V^ males exhibited longer escape times at days 2 and 3 compared with Cx30^+/+^ and Cx30^A88V/+^ mice (Fig. 8C). On day 4, however, male homozygous mutant mice demonstrated escape times similar to those of WT and heterozygous mutants (Fig. 8C). In female mice, both WT and heterozygous mutants exhibited decreasing escape time over the 4 days of testing (Fig. 8D), whereas homozygous mutants did not show any evidence of learning (Fig. 8D). During the spatial learning task, all mice demonstrated similar swimming speeds, suggesting that delayed learning or lack of learning was not attributable to poor swimming performance (Fig. 8E,F).

## DISCUSSION

At present, no homozygous *GJB6* missense mutations have been identified in the human population. However, heterozygous *GJB6* missense mutations do exist in patient cohorts, with variable pathological manifestations that most often affect hearing and skin health, but it is not at all clear whether and how *GJB6* mutations affect the brain, where Cx30 is widely expressed (Lamartine et al., 2000; Tan and Tay, 2000). Mutant mice lacking Cx30 demonstrate reduced exploratory activity, with evidence of increased anxiety-like behavior (Dere et al., 2003), providing a hint that Cx30 might be linked to cognitive functions. To interrogate the nature and breadth of how Cx30 mutations cause disease, a genetically modified mouse line was generated, in which Cx30 p.A88V was expressed in both the homozygous and heterozygous context (Bosen et al., 2014). The *GJB6* mutation that results in Cx30 p.A88V is best known to cause HED2, which manifests clinically as poor skin health (Lamartine et al., 2000; Smith et al., 2002). Given the potential impact of this *GJB6* mutation on the brain, where Cx30 is known to be expressed in a variety of cell types, we used both heterozygous and homozygous 3- to 6-month-old Cx30-A88V mutant mice (Bosen et al., 2014) to characterize the effect of the A88V mutant on the brain and potential impact on behavioral performance. Furthermore, we investigated both male and female mouse cohorts to assess whether there were unique sex-dependent differences.

Examination of brain morphology revealed hydrocephalus in Cx30^A88V/A88V^ mice, in which the brain weight and ventricles were enlarged in male and female mice compared with heterozygous and WT groups. In male mouse brains, Cx30 levels were lowest in Cx30^A88V/A88V^ mice compared with the other genotypes. In female brains, however, both Cx30^A88V/+^ and Cx30^A88V/A88V^ mice demonstrated lower brain levels of Cx30 compared with WT mice, although it was not possible to assign this decrease to any one specific cell type. Interestingly, only female mutant mouse brain tissue exhibited lower levels of Cx43, suggesting that females might have a more severe phenotype, which received support from our behavioral studies. Only female Cx30^A88V/A88V^ mice demonstrated greater exploratory behavior in the new environment. Female Cx30^A88V/A88V^ mice were also the only group to spend a greater proportion of time in the open arm of the elevated plus maze. Although the elevated plus maze can report on anxiety-like behavior, it is likely that the phenotype in this task is more related to deficits in innate behavior to recognize the threat of the open arm. Both male and female Cx30^A88V/A88V^ mice performed less efficiently in the cued learning task compared with heterozygous and WT groups. These results might suggest that their vision or ability to detect visual cues could also be impaired. Nonetheless, homozygous mutant mice were still able to find the platform, albeit taking a longer time. Remarkably, in the spatial learning test, female Cx30^A88V/A88V^ mice were unable to learn the location of the hidden platform during the acquisition phase. Overall, our findings indicate that the Cx30-A88V mutant impacts the development and health of the male and female mouse brain, but female mice exhibit far greater cognitive deficits.

In the brain, Cx30 is found in astrocytes, but to a much lesser extent than Cx43 (Charvériat et al., 2017). Cx30 is also expressed in the ependymal cell layer separating the brain parenchyma from the cerebral spinal fluid (CSF) space of the ventricles and in leptomeningeal cells surrounding blood vessels in the subarachnoid space (De Bock et al., 2014). Interestingly, these tissues engage in CSF production and circulation (Brinker et al., 2014), which might explain, in part, the hydrocephalus phenotype seen in aging Cx30^A88V/A88V^ mice. Using cellular expression models, our group and others have previously shown that the Cx30-A88V mutant is cytotoxic when ectopically expressed in HeLa cells and keratinocytes (Berger et al., 2014; Essenfelder et al., 2004; Lu et al., 2018), most probably owing to its assembly into leaky hemichannels (Essenfelder et al., 2004; Kuang et al., 2020). This raises the possibility that the homozygous expression of the A88V mutant is causing ependymal cells to malfunction. Ependymal cells are typically coupled via gap junctions with one another and with the surrounding glial cells (Brinker et al., 2014; De Bock et al., 2014). Although the ependymal cell layer of homozygous mutant mice appeared unorganized, Cx43 was still readily observed, suggesting that this cell layer was still somewhat intact, although Cx30 levels were low. We suspect that low Cx30 function might have compounding disruptive effects on normal CSF production and flow in the brain, resulting in hydrocephalus. Astrocytes have also been implicated in governing CSF fluid exchange, in addition to choroid plexus CSF production (Brinker et al., 2014; De Bock et al., 2014), because their processes are interspersed in the ependymal layer and tethered to brain capillaries. In related studies, Cx30/Cx43 astroglial knockout mice also demonstrate reduced aquaporin 4 levels in the brain (Ezan et al., 2012), altering transmembrane water flux (Simard et al., 2003). Although morphological brain anomalies have not been reported in HED2 patients, on occasion patients with connexin 26 (Cx26; also known as *GJB2* when referring to the gene)-linked keratitis-ichthiosis-deafness (KID) syndrome present with Dandy–Walker malformation resulting in cystic dilatation of the fourth ventricle and hydrocephalus (Todt et al., 2009), linking connexin status to CSF homeostasis.

Cx30-deficient mice have been characterized in behavioral studies to avoid open field areas (Dere et al., 2003), whereas mice harboring Cx43-deficient astrocytes exhibit greater exploratory behavior (Frisch et al., 2003; Theis et al., 2003). We found that only female Cx30^A88V/A88V^ mice demonstrated greater locomotive activity in the open field test, with a preference for exploring the open arm of the elevated plus maze to the point of even peering over the end of the arm. The reason for this same behavior not occurring in male homozygous mutant mice is not clear, but might be related to the observed reduction in Cx43 expression levels in females. This alteration might affect connection to astrocytes that are extensively coupled by Cx43 gap junctions. The conserved expression of Cx43 in all male genotypes might be enough to sustain astrocyte-dependent communication mechanisms affecting behavioral patterns, regardless of reduced functional Cx30 in the brain of Cx30^A88V/A88V^ mice. The reason why any A88V mutant mice have increased exploratory curiosity, whereas Cx30 null mice have less, remains to be determined but might be related to crosstalk between the Cx30 mutant and downstream signaling molecules, activated pathways that would be lacking in Cx30 null mice. In fact, mice conditionally lacking Cx26 from neocortical excitatory neurons exhibited elevated anxiety that might be linked to impaired development of the neocortex (Su et al., 2017). We also cannot discount the noted changes in anxiety-like behavior and curiosity that might be linked to potential crosstalk between the Cx30-A88V mutant and Cx26, because both are co-expressed in leptomeningeal cells (Lynn et al., 2011; Nagy et al., 2001). Collectively, these studies reveal the complexities of how loss of connexins or mutant connexins might be regulating mouse anxiety and curiosity (Del Bigio, 2010a,b).

Finally, spatial learning was tested with the Morris water maze, and female homozygous mutant mice yet again exhibited poorer performance than males, although both showed deficits in the ability to learn. Although all other experimental groups learned the spatial acquisition task during 4 days of testing, female homozygous mutant mice demonstrated less capacity to learn. Even during the cued learning trials, the female homozygous mutant mice tended to take longer to find the marked platform compared with other groups, potentially suggesting that they might have visual and cue-detection impairments.

In summary, both male and female Cx30^A88V/A88V^ mice exhibited hydrocephaly and enlarged ventricles accompanied by variable and sex-dependent reductions in Cx30 and/or Cx43 levels. Sex differences were also evident, because female homozygous mutant mice presented with a range of behavioral alterations. Whether these alterations occur because female mice are less resilient to the ventricular dysfunction or owing to the compound effect of Cx43 reduction observed in females remains to be determined. Importantly, both male and female heterozygous mutant mice, which model autosomal dominant HED2, did not present with major brain structural or behavioral changes. These results suggest that heterozygous *GJB6* mutations might not cause enough deficits to affect cognitive function in humans. By contrast, the major brain phenotypes and the sex-specific connexin differences in mutant mouse brains impacting behavioral outcomes might contribute to the lack of homozygous *GJB6* missense mutations in the human population. Nevertheless, our study firmly adds to the growing body of evidence that connexins found in the brain might play crucial roles in cognitive function.

## MATERIALS AND METHODS

### Mice

Cx30^A88V/+^ and Cx30^A88V/A88V^ mice were generated as previously described (Bosen et al., 2014) and kindly provided by Dr Klaus Willecke (Life and Medical Sciences Institute, Bonn, Germany), bred in a CD-1 background, and genotyped as previously described (Bosen et al., 2014; Kelly et al., 2019; Lukashkina et al., 2017). Three- to 6-month-old male and female Cx30^+/+^ (noted as WT in figures), Cx30^A88V/+^ and Cx30^A88V/A88V^ mice were used in littermate-controlled experiments and housed in the animal care facilities at the University of Western Ontario. Mice were provided with unlimited food and water and maintained in the dark for 12 h each day. Mice were euthanized via CO_2_ asphyxiation. In keeping with the Canadian Council of Animal Care, all studies were reviewed and approved by the Animal Care Committee at the University of Western Ontario (Protocol 2015-030; 2019-009).

### Histology

Brains fixed in 4% paraformaldehyde (PFA) were embedded in paraffin, and 5- to 10-µm-thick sections were collected at 100 µm intervals. After staining with Hematoxylin and Eosin, brain sections were imaged. For all mouse groups (*n*=3 cohorts), brain ventricle areas were measured using ImageJ and the data expressed relative to the total cross-sectional area of the brain.

### qPCR analysis

RNA was collected using Qiagen RNeasy kits (Qiagen) from dissected and flash-frozen whole brains of 3- to 6-month-old male and female mice. Complementary DNA (cDNA) was produced using the first-strand cDNA synthesis kit (SuperScript VI LO; Thermo Fisher Scientific). Transcript levels were analyzed using mouse-specific primers (*Cx43*, 5′-AAATGTCTGCTATGACAAGTCCTTC-3′ and 5′-CTTTGAGCTCCTCTTCTTTCTTGTT-3′; *Cx30*, 5′-GGCCGAGTTGTGTTACCTGCT-3′ and 5′-TCTCTTTCAGGGCATGGTTGG-3′; and *Gapdh*, 5′-CGACTTCAACAGCAACTCCCACTCTTCC-3′, 5′-TGGGTGGTCCAGGGTTTCTTACTCCTT-3′) and the PowerUp SYBR Green Mastermix (Thermo Fisher Scientific) in a Bio-Rad CFX96 real-time system. Transcripts were normalized to GAPDH mRNA. Normalized mRNA expression levels were analyzed using the ΔΔ*C_T_* method, which was calculated using Bio-Rad software. A WT mouse sample was set as the control for all calculations, and data were expressed relative to this sample (*n*=5-7 per group for males, *n*=4-8 per group for females).

### Western blotting

Mouse brain tissue lysates were prepared on ice via homogenization of the whole brain in lysis buffer (150 mM NaCl, 1 mM EDTA, 1 mM EGTA, 1% Triton X-100 and 10 mM Tris-HCl) containing protease and phosphatase inhibitors (Roche-Applied Sciences; 100 mM NaF and 100 mM Na_3_VO_4_). Thirty microgram samples of protein from tissues lysates were resolved on a 10% SDS-PAGE gel and transferred nitrocellulose membranes using an iBlot Dry Blotting system (Invitrogen). Membranes were blocked in 3% bovine serum albumin-PBS (blocking solution) for 30 min at room temperature. Membranes were immunolabeled using the following primary antibodies: rabbit anti-Cx43 (1:5000; Sigma-Aldrich; C6219); rabbit anti-Cx30 (1:300; Thermo Fisher Scientific; 71-2200) and mouse anti-GAPDH (1:10,000; Santa Cruz Biotechnology; sc-365062), diluted in blocking solution at 4°C overnight. Membranes were washed three times, for 5 min each time, with PBS containing 0.5% Tween 20 and incubated with fluorescence-tagged secondary anti-rabbit Alexa Fluor 680 (1:10,000; LI-COR Biosciences; ab175772) or anti-mouse IRdye 800 (1:10,000; Rockland Immunochemicals; 610-132-003). Quantification of protein expression was performed using an Odyssey Infrared Imaging System and accompanying software for densitometry analysis (LI-COR Biosciences). Samples were quantified after normalization to GAPDH loading controls (*n*=5-7 per group for males, *n*=5-8 per group for females).

### Immunofluorescence microscopy

Male and female mouse brains were dissected and immersed in 10% formalin at 4°C for ∼48 h, washed in PBS, immersed in 30% sucrose and maintained at 4°C for cryopreservation. Brains were embedded in an agarose solution, flash-frozen and left to acclimate in a cryostat (Leica Biosystems, Wetzlar, Germany) at −25°C for ∼30 min. Using the Allen Brain Atlas as a guide (https://mouse.brain-map.org/), coronal brain sections were taken between ∼4000 µm and ∼7000 µm caudal to the front of the brain to account for variability in cryosection slide mounting. Sections were cut at 14 µm thickness, mounted on glass slides and stored at −20°C until use. Immunofluorescence labeling was performed on coronal brain sections where lateral ventricles could be identified. Brain sections were circled with ImmEdge hydrophobic barrier pen (VectorLabs, Burlingame, CA, USA), then blocked and permeabilized in 3% bovine serum albumin+0.2% Triton X-100 solution for 1 h at room temperature. Samples were incubated with the following primary antibodies overnight at 4°C: rabbit anti-Cx43 (1:750; Sigma-Aldrich; C6219), rabbit anti-Cx30 (1:400; Thermo Fisher Scientific; 71-2200) and mouse anti-GFAP (1:200; Sigma-Aldrich; 63893). Samples were then washed and incubated with an Alexa Fluor fluorescence-conjugated secondary antibody for 1 h at room temperature, followed by Hoechst 33342 nuclear stain (1:10,000; Invitrogen). Samples were mounted with coverslips and fluorescence images acquired using a Zeiss LSM 800 laser scanning confocal microscope.

### Experimental design of behavioral studies

Cognitive and sensorimotor function was assessed in two cohorts of mice (57 total, minimum of eight mice from each genotype/sex grouping). Experiments were performed in the following order: open field test, elevated plus maze and spatial and cued Morris water maze. Mice aged 3-6 months were housed with littermates when possible in the rodent neurobehavioral core facility at Robarts Research Institute for at least 1 week before testing. Each day, mice were brought to the laboratory and left unattended in their home cages for at least 20 min before testing. All experiments were performed by the same experimenter between 09.00 and 17.00, during the light phase of the light-dark cycle.

### Open field test

General locomotor activity was assessed as previously described by Zubrycki et al. (1990). Briefly, mice were placed in diagonally opposite 20×20 cm quadrants of a square arena and left to explore the enclosure freely in the absence of the experimenter. For 30 min, locomotor activity in the *x*-, *y*- and *z*-axes was recorded by the infrared beam detectors of open field locomotor boxes (Omnitech Electronics, Columbus, OH, USA), and data were acquired using VersaMax software (Omnitech Electronics). Enclosure surfaces were thoroughly cleaned with 70% ethanol before each trial to remove debris and scent cues. For each box, two mice were recorded simultaneously, and genotype/sex group pairings were balanced to minimize potential group interactions.

### Elevated plus maze

Exploratory behaviors were assessed as previously described by Martins-Silva et al. (2011). Briefly, mice were placed on an elevated white ‘+’-shaped platform with opposing open arms perpendicular to opposing closed arms. Closed arms were shaded by ∼20-cm-high black plastic walls. Mice were placed individually in the middle of the maze facing a closed arm and left to explore freely for 5 min. Video monitoring recorded the position of the animal to ANY-maze software (Wood Dale, IL, USA), and the percentages of time mice spent in the center, open and closed arms were calculated. Maze surfaces were thoroughly cleaned with 70% ethanol before each trial to remove debris and scent cues.

### Morris water maze

The spatial and cued water maze tests were performed as previously described by Vorhees and Williams (2006). Briefly, mice were placed initially in individual test cages for 5 min before testing. The spatial learning phase consisted of lowering mice into a 1.2-m-diameter water bath at one of four positions and leaving them to explore the bath for up to 90 s. Mice that failed to find the clear target platform submerged 1.5 cm below the water surface were guided towards it and left on the platform for 15 s before returning to their test cage. Four up-lights placed near the bath lit the testing room. The water temperature was maintained at 25°C, and prominent visual cues (black and white patterns) were located on the walls around the water bath. Each animal performed four trials per day, in which they were lowered into the bath at a different initial position. Mice had a minimum of 30 min to rest between trials and were tested on four consecutive days. Video monitoring tracked the position of the animal in the water bath, and the time to escape the maze (finding and climbing onto the platform) was recorded for each trial. An average escape time was calculated for each mouse on each day of spatial acquisition. After the spatial test, a cued learning test was performed, wherein mice were tested in an identical manner to the spatial acquisition task, with the addition of a prominent visual cue indicating the position of the hidden platform (15 cm-tall orange and black flag). The time to escape was recorded and averaged over four trials. The target platform position, order of initial positions and order of animal testing were balanced by genotype/sex groupings.

### Statistics

Results are provided as means±s.e.m. and compared between genotypes within male and female cohorts to evaluate sex as a biological variable. One-way ANOVA with Tukey's post hoc test was used to determine changes between genotype means for mRNA and protein expression, body and brain weight, total distance traveled in the open field test and cued learning escape time in the Morris water maze. If the Shapiro–Wilk test for normality deemed genotype data non-normally distributed, a non-parametric Kruskal–Wallis test with Dunn's multiple comparisons was performed on genotype means. Two-way ANOVA with Tukey's post hoc test was used to compare genotype means for time spent in the open and closed arms of the elevated plus maze. Two-way repeated-measures ANOVA with Tukey's post hoc test was used to compare escape time and swimming speed means for the spatial learning phase of the Morris water maze. All statistical analyses were achieved using Prism v.8 (GraphPad, La Jolla, CA, USA). Means were considered statistically significant when *P*<0.05.

## References

[DMM046235C1] Abudara, V., Bechberger, J., Freitas-Andrade, M., De Bock, M., Wang, N., Bultynck, G., Naus, C. C., Leybaert, L. and Giaume, C. (2014). The connexin43 mimetic peptide Gap19 inhibits hemichannels without altering gap junctional communication in astrocytes. *Front. Cell Neurosci.* 8, 306 10.3389/fncel.2014.00306 25374505 PMC4204617

[DMM046235C2] Avshalumova, L., Fabrikant, J. and Koriakos, A. (2013). Overview of skin diseases linked to connexin gene mutations. *Int. J. Dermatol.* 53, 192-205. 10.1111/ijd.12062 23675785

[DMM046235C3] Baris, H. N., Zlotogorski, A., Peretz-Amit, G., Doviner, V., Shohat, M., Reznik-Wolf, H. and Pras, E. (2008). A novel GJB6 missense mutation in hidrotic ectodermal dysplasia 2 (Clouston syndrome) broadens its genotypic basis. *Br. J. Dermatol.* 159, 1373-1376. 10.1111/j.1365-2133.2008.08796.x 18717672

[DMM046235C4] Berger, A. C., Kelly, J. J., Lajoie, P., Shao, Q. and Laird, D. W. (2014). Mutations in Cx30 that are linked to skin disease and non-syndromic hearing loss exhibit several distinct cellular pathologies. *J. Cell Sci.* 127, 1751-1764. 10.1242/jcs.138230 24522190

[DMM046235C5] Bosen, F., Schütz, M., Beinhauer, A., Strenzke, N., Franz, T. and Willecke, K. (2014). The Clouston syndrome mutation connexin30 A88V leads to hyperproliferation of sebaceous glands and hearing impairments in mice. *FEBS Lett.* 588, 1795-1801. 10.1016/j.febslet.2014.03.040 24685692

[DMM046235C6] Brinker, T., Stopa, E., Morrison, J. and Klinge, P. (2014). A new look at cerebrospinal fluid circulation. *Fluids Barriers CNS* 11, 10 10.1186/2045-8118-11-10 24817998 PMC4016637

[DMM046235C7] Charvériat, M., Naus, C. C., Leybaert, L., Sáez, J. C. and Giaume, C. (2017). Connexin-dependent neuroglial networking as a new therapeutic target. *Front. Cell Neurosci.* 11, 174 10.3389/fncel.2017.00174 28694772 PMC5483454

[DMM046235C8] Common, J. E. A., Becker, D., Di, W.-L., Leigh, I. M., O'Toole, E. A. and Kelsell, D. P. (2002). Functional studies of human skin disease- and deafness-associated connexin 30 mutations. *Biochem. Biophys. Res. Commun.* 298, 651-656. 10.1016/S0006-291X(02)02517-2 12419304

[DMM046235C9] De Bock, M., Vandenbroucke, R. E., Decrock, E., Culot, M., Cecchelli, R. and Leybaert, L. (2014). A new angle on blood-CNS interfaces: a role for connexins? *FEBS Lett.* 588, 1259-1270. 10.1016/j.febslet.2014.02.060 24631535

[DMM046235C10] Del Bigio, M. R. (2010a). Ependymal cells: biology and pathology. *Acta Neuropathol.* 119, 55-73. 10.1007/s00401-009-0624-y 20024659

[DMM046235C11] Del Bigio, M. R. (2010b). Neuropathology and structural changes in hydrocephalus. *Dev. Disabil. Res. Rev.* 16, 16-22. 10.1002/ddrr.94 20419767

[DMM046235C12] Dere, E., De Souza-Silva, M. A., Frisch, C., Teubner, B., Sohl, G., Willecke, K. and Huston, J. P. (2003). Connexin30-deficient mice show increased emotionality and decreased rearing activity in the open-field along with neurochemical changes. *Eur. J. Neurosci.* 18, 629-638. 10.1046/j.1460-9568.2003.02784.x 12911759

[DMM046235C13] Di, W.-L., Rugg, E. L., Leigh, I. M. and Kelsell, D. P. (2001). Multiple epidermal connexins are expressed in different keratinocyte subpopulations including connexin 31. *J. Invest. Dermatol.* 117, 958-964. 10.1046/j.0022-202x.2001.01468.x 11676838

[DMM046235C14] Essenfelder, G. M., Bruzzone, R., Lamartine, J., Charollais, A., Blanchet-Bardon, C., Barbe, M. T., Meda, P. and Waksman, G. (2004). Connexin30 mutations responsible for hidrotic ectodermal dysplasia cause abnormal hemichannel activity. *Hum. Mol. Genet.* 13, 1703-1714. 10.1093/hmg/ddh191 15213106

[DMM046235C15] Ezan, P., André, P., Cisternino, S., Saubaméa, B., Boulay, A.-C., Doutremer, S., Thomas, M.-A., Quenech'du, N., Giaume, C. and Cohen-Salmon, M. (2012). Deletion of astroglial connexins weakens the blood-brain barrier. *J. Cereb. Blood Flow Metab.* 32, 1457-1467. 10.1038/jcbfm.2012.45 22472609 PMC3421093

[DMM046235C16] Forge, A., Becker, D., Casalotti, S., Edwards, J., Marziano, N. and Nevill, G. (2003). Gap junctions in the inner ear: comparison of distribution patterns in different vertebrates and assessement of connexin composition in mammals. *J. Comp. Neurol.* 467, 207-231. 10.1002/cne.10916 14595769

[DMM046235C17] Fraser, F. C. and Der Kaloustian, V. M. (2001). A man, a syndrome, a gene: Clouston's hidrotic ectodermal dysplasia (HED). *Am. J. Med. Genet.* 100, 164-168. 10.1002/1096-8628(20010422)100:2<164::AID-AJMG1244>3.0.CO;2-W 11298380

[DMM046235C18] Frisch, C., Theis, M., De Souza Silva, M. A., Dere, E., Sohl, G., Teubner, B., Namestkova, K., Willecke, K. I. and Huston, J. P. (2003). Mice with astrocyte-directed inactivation of connexin43 exhibit increased exploratory behaviour, impaired motor capacities, and changes in brain acetylcholine levels. *Eur. J. Neurosci.* 18, 2313-2318. 10.1046/j.1460-9568.2003.02971.x 14622192

[DMM046235C19] Goldberg, G. S., Moreno, A. P. and Lampe, P. D. (2002). Gap junctions between cells expressing connexin 43 or 32 show inverse permselectivity to adenosine and ATP. *J. Biol. Chem.* 277, 36725-36730. 10.1074/jbc.M109797200 12119284

[DMM046235C20] Goodenough, D. A. and Paul, D. L. (2003). Beyond the gap: functions of unpaired connexon channels. *Nat. Rev. Mol. Cell Biol.* 4, 285-294. 10.1038/nrm1072 12671651

[DMM046235C21] Jan, A. Y., Amin, S., Ratajczak, P., Richard, G. and Sybert, V. P. (2004). Genetic heterogeneity of KID syndrome: identification of a Cx30 gene (GJB6) mutation in a patient with KID syndrome and congenital atrichia. *J. Invest. Dermatol.* 122, 1108-1113. 10.1111/j.0022-202X.2004.22518.x 15140211

[DMM046235C22] Kelly, J. J., Simek, J. and Laird, D. W. (2015). Mechanisms linking connexin mutations to human diseases. *Cell Tissue Res.* 360, 701-721. 10.1007/s00441-014-2024-4 25398718

[DMM046235C23] Kelly, J. J., Abitbol, J. M., Hulme, S., Press, E. R., Laird, D. W. and Allman, B. L. (2019). The connexin 30 A88V mutant reduces cochlear gap junction expression and confers long-term protection against hearing loss. *J. Cell Sci.* 132, jcs224097 10.1242/jcs.224097 30559251

[DMM046235C24] Kuang, Y., Zorzi, V., Buratto, D., Ziraldo, G., Mazzarda, F., Peres, C., Nardin, C., Salvatore, A. M., Chiani, F., Scavizzi, F. et al. (2020). A potent antagonist antibody targeting connexin hemichannels alleviates Clouston syndrome symptoms in mutant mice. *EBioMedicine* 57, 102825 10.1016/j.ebiom.2020.102825 32553574 PMC7378960

[DMM046235C25] Kunzelmann, P., Schroder, W., Traub, O., Steinhauser, C., Dermietzel, R. and Willecke, K. (1999). Late onset and increasing expression of the gap junction protein connexin30 in adult murine brain and long-term cultured astrocytes. *Glia* 25, 111-119. 10.1002/(SICI)1098-1136(19990115)25:2<111::AID-GLIA2>3.0.CO;2-I 9890626

[DMM046235C26] Laird, D. W. (2006). Life cycle of connexins in health and disease. *Biochem. J.* 394, 527-543. 10.1042/BJ20051922 16492141 PMC1383703

[DMM046235C27] Laird, D. W. (2008). Closing the gap on autosomal dominant connexin-26 and connexin-43 mutants linked to human disease. *J. Biol. Chem.* 283, 2997-3001. 10.1074/jbc.R700041200 18089569

[DMM046235C28] Laird, D. W. and Lampe, P. D. (2018). Therapeutic strategies targeting connexins. *Nat. Rev. Drug Discov.* 17, 905-921. 10.1038/nrd.2018.138 30310236 PMC6461534

[DMM046235C29] Laird, D. W., Naus, C. C. and Lampe, P. D. (2017). SnapShot: connexins and disease. *Cell* 170, 1260-1260.e1. 10.1016/j.cell.2017.08.034 28886388

[DMM046235C30] Lamartine, J., Munhoz Essenfelder, G., Kibar, Z., Lanneluc, I., Callouet, E., Laoudj, D., Lemaitre, G., Hand, C., Hayflick, S. J., Zonana, J. et al. (2000). Mutations in GJB6 cause hidrotic ectodermal dysplasia. *Nat. Genet.* 26, 142-144. 10.1038/79851 11017065

[DMM046235C31] Leybaert, L., Lampe, P. D., Dhein, S., Kwak, B. R., Ferdinandy, P., Beyer, E. C., Laird, D. W., Naus, C. C., Green, C. R. and Schulz, R. (2017). Connexins in cardiovascular and neurovascular health and disease: pharmacological implications. *Pharmacol. Rev.* 69, 396-478. 10.1124/pr.115.012062 28931622 PMC5612248

[DMM046235C32] Lu, Y., Zhang, R., Wang, Z., Zhou, S., Song, Y., Chen, L., Chen, N., Liu, W., Ji, C., Wu, W. et al. (2018). Mechanistic effect of the human GJB6 gene and its mutations in HaCaT cell proliferation and apoptosis. *Braz. J. Med. Biol. Res.* 51, e7560 10.1590/1414-431x20187560 30043857 PMC6065815

[DMM046235C33] Lukashkina, V. A., Levic, S., Lukashkin, A. N., Strenzke, N. and Russell, I. J. (2017). A connexin30 mutation rescues hearing and reveals roles for gap junctions in cochlear amplification and micromechanics. *Nat. Commun.* 8, 14530 10.1038/ncomms14530 28220769 PMC5321796

[DMM046235C34] Lynn, B. D., Tress, O., May, D., Willecke, K. and Nagy, J. I. (2011). Ablation of connexin30 in transgenic mice alters expression patterns of connexin26 and connexin32 in glial cells and leptomeninges. *Eur. J. Neurosci.* 34, 1783-1793. 10.1111/j.1460-9568.2011.07900.x 22098503

[DMM046235C35] Martins-Silva, C., De Jaeger, X., Guzman, M. S., Lima, R. D. F., Santos, M. S., Kushmerick, C., Gomez, M. V., Caron, M. G., Prado, M. A. M. and Prado, V. F. (2011). Novel strains of mice deficient for the vesicular acetylcholine transporter: insights on transcriptional regulation and control of locomotor behavior. *PLoS ONE* 6, e17611 10.1371/journal.pone.0017611 21423695 PMC3053374

[DMM046235C36] Mazaré, N., Gilbert, A., Boulay, A.-C., Rouach, N. and Cohen-Salmon, M. (2018). Connexin 30 is expressed in a subtype of mouse brain pericytes. *Brain Struct. Funct.* 223, 1017-1024. 10.1007/s00429-017-1562-4 29143947

[DMM046235C37] Nagy, J. I., Li, X., Rempel, J., Stelmack, G., Patel, D., Staines, W. A., Yasumura, T. and Rash, J. E. (2001). Connexin26 in adult rodent central nervous system: demonstration at astrocytic gap junctions and colocalization with connexin30 and connexin43. *J. Comp. Neurol.* 441, 302-323. 10.1002/cne.1414 11745652

[DMM046235C38] Simard, M., Arcuino, G., Takano, T., Liu, Q. S. and Nedergaard, M. (2003). Signaling at the gliovascular interface. *J. Neurosci.* 23, 9254-9262. 10.1523/JNEUROSCI.23-27-09254.2003 14534260 PMC6740832

[DMM046235C39] Smith, F. J. D., McLean, W. H. I. and Morley, S. M. (2002). A novel connexin 30 mutation in Clouston syndrome. *J. Invest. Dermatol.* 118, 530-532. 10.1046/j.0022-202x.2001.01689.x 11874494

[DMM046235C40] Sohl, G. and Willecke, K. (2004). Gap junctions and the connexin protein family. *Cardiovasc. Res.* 62, 228-232. 10.1016/j.cardiores.2003.11.013 15094343

[DMM046235C41] Srinivas, M., Verselis, V. K. and White, T. W. (2018). Human diseases associated with connexin mutations. *Biochim. Biophys. Acta* 1860, 192-201. 10.1016/j.bbamem.2017.04.024 PMC5659969 28457858

[DMM046235C42] Su, X., Chen, J.-J., Liu, L.-Y., Huang, Q., Zhang, L.-Z., Li, X.-Y., He, X.-N., Lu, W., Sun, S., Li, H. et al. (2017). Neonatal CX26 removal impairs neocortical development and leads to elevated anxiety. *Proc. Natl. Acad. Sci. USA* 114, 3228-3233. 10.1073/pnas.1613237114 28265099 PMC5373364

[DMM046235C43] Tan, E. and Tay, Y.-K. (2000). What syndrome is this? Hidrotic ectodermal dysplasia (Clouston syndrome). *Pediatr. Dermatol.* 17, 65-67. 10.1046/j.1525-1470.2000.01713.x 10720992

[DMM046235C44] Theis, M., Jauch, R., Zhuo, L., Speidel, D., Wallraff, A., Döring, B., Frisch, C., Sohl, G., Teubner, B., Euwens, C. et al. (2003). Accelerated hippocampal spreading depression and enhanced locomotory activity in mice with astrocyte-directed inactivation of connexin43. *J. Neurosci.* 23, 766-776. 10.1523/JNEUROSCI.23-03-00766.2003 12574405 PMC6741919

[DMM046235C45] Todt, I., Mazereeuw-Hautier, J., Binder, B. and Willems, P. J. (2009). Dandy-Walker malformation in patients with KID syndrome associated with a heterozygote mutation (p.Asp50Asn) in the GJB2 gene encoding connexin 26. *Clin. Genet.* 76, 404-408. 10.1111/j.1399-0004.2009.01211.x 19793313

[DMM046235C46] Vorhees, C. V. and Williams, M. T. (2006). Morris water maze: procedures for assessing spatial and related forms of learning and memory. *Nat. Protoc.* 1, 848-858. 10.1038/nprot.2006.116 17406317 PMC2895266

[DMM046235C47] Zhan, Y., Luo, S., Pi, Z. and Zhang, G. (2020). A recurrent mutation of GJB6 in a big Chinese family with Hidrotic ectodermal dysplasia. *Hereditas* 157, 34 10.1186/s41065-020-00148-8 32843087 PMC7446134

[DMM046235C48] Zhang, X.-J., Chen, J.-J., Yang, S., Cui, Y., Xiong, X.-Y., He, P.-P., Dong, P.-L., Xu, S.-J., Li, Y.-B., Zhou, Q. et al. (2003). A mutation in the connexin 30 gene in Chinese Han patients with hidrotic ectodermal dysplasia. *J. Dermatol. Sci.* 32, 11-17. 10.1016/S0923-1811(03)00033-1 12788524

[DMM046235C49] Zubrycki, E. M., Giordano, M. and Sanberg, P. R. (1990). The effects of cocaine on multivariate locomotor behavior and defecation. *Behav. Brain Res.* 36, 155-159. 10.1016/0166-4328(90)90169-F 2302315

